# A population-based cross-sectional study on the situation of cervical cancer screening in Liaoning, China

**DOI:** 10.1186/s12905-023-02249-8

**Published:** 2023-03-29

**Authors:** Bo Zhu, Huihui Yu, Ping Ni, Xi Chen, Jing Zhang, Danbo Wang

**Affiliations:** 1 Department of Cancer prevention and treatment, Cancer Hospital of China Medical University, Liaoning Cancer Hospital & Institute, Shenyang, Liaoning Province China; 2 Department of Epidemiology, Cancer Hospital of China Medical University, Liaoning Cancer Hospital & Institute, Shenyang, Liaoning Province China; 3 Department of Gynaecology, Cancer Hospital of China Medical University, Liaoning Cancer Hospital & Institute, No. 44 Xiaoheyan Road, Dadong District Shenyang, 110001 Shenyang, Liaoning Province China

**Keywords:** Cervical cancer, Screening, Willingness, Population-based

## Abstract

**Background:**

Cervical cancer (CC) screening is currently recognized as an effective intervention for CC. Previous studies found that the proportion of screening was low in China, especially in Liaoning. Therefore, we performed a population-based cross-sectional survey to investigate the situation of cervical cancer screening and analyze their related factors for providing a decision-making basis for sustainable and effective development of cervical cancer screening.

**Methods:**

This population-based cross-sectional study involved aged 30 to 69 years in nine counties/districts in Liaoning from 2018 to 2019. Data were collected using the quantitative data collection methods, and analyzed in SPSS version 22.0.

**Results:**

Overall, only 22.37% of 5334 respondents reported having ever been screened for cervical cancer in past 3 years, and 38.41% of respondents reported having the willingness for cervical cancer screening in next 3 years. In the rate of CC screening, multilevel analysis indicated that age, marital status, education level, type of occupation, medical insurance, family income, place of residence and regional economic level had a significant impact on proportion of screening. In the rate of CC screening willingness, multilevel analysis indicated that age, family income, health status, place of residence, regional economic level and CC screening still had a significant impact, but marital status, education level and medical insurance type had no significant impact. There was no significant difference in marital status, education level and medical insurance type after the factors of CC screening were added in the model.

**Conclusion:**

Our study found both proportion of screening and willingness were at a low level, and age, economic and regional factors were the main factors for implementation of CC screening in China. In the future, targeted policies should be formulated according to the characteristics of different groups of people, and reduce the gap in the current health service capacity between different regions.

## Introduction

Cervical cancer (CC) ranks as the fourth most frequently diagnosed cancer and the fourth leading cause of cancer death in women[1]. In China, the incidence and mortality of CC is increasing by the growing average life expectancy. The incidence rate in 2015 of 16.56 per 100,000 was 1.43 times higher than in 2014, the mortality rate in 2015 of 3.12 per 100,000 was 1.62 times higher than in 2014, and the disease burden of CC increased[2, 3]. World Health Organization (WHO) established that CC should no longer be considered a public health problem when the age-adjusted incidence rate is less than 4 per 100,000. Although incidence cannot be reduced to zero with the current interventions, the elimination threshold is achievable within the 21st century in every country[4]. Therefore, WHO have a highly ambitious plan for CC elimination in 2030, as the largest developing country, China bears an important responsibility. Due to vast territory, large population and the unbalanced social and economic development level among different regions, the prevention and control of CC in China is facing great difficulties[5]. Therefore, it is necessary to take targeted measures and strategies to improve the goal of CC screening.

In the past decade, China always paid attention to the prevention and control of CC. CC screening is currently recognized as an effective intervention to prevent the occurrence and death of CC[6]. Even in high-income countries where HPV vaccine has been widely used, CC screening is still a conventional way of prevention. As a part of this health care reform, China’s government launched a major public health service project, namely, the National Cervical Cancer Screening Program (NCCSP)[7]. The NCCSP began in 2009, and was jointly promoted by the National Health Administration and the All-China Women’s Federation to carry out free screening for women aged 30–59 (adjusted to 35–64 in 2012)[8]. The traditional Pap’s method of cervical cytology was used for the initial screening, and the subjects with positive results continued to undergo colposcopy and pathological examination. The local maternal and child health and family planning service centers are specifically responsible for organizing the implementation[8]. Generally, the nurse samples the vaginal secretion, the pathologist judges the cytological results, and the colposcopy is performed by the doctor. All data information registered and entered the information platform. In 2012, WHO recommended “Percentage of women between the ages of 30–49 screened for CC at least once, or more often, and for lower or higher age groups according to national programs or policies.” as an indicators in NCD Global Monitoring Framework[9]. CC proportion of screening is the most direct indicator reflecting the effect of CC screening services, and improving proportion of screening is the prerequisite for successful prevention and control of CC.

Liaoning is a provincial administrative region located in the northeast of China. The data from Liaoning provincial Bureau of Statistics showed that the population living in cities and rural areas were 30.7 million and 11.9 million, respectively, and basic medical insurance covered more than 89% of people in Liaoning Province in 2020[10]. There are three social health insurance schemes with different target population, financing and reimbursement rate: The Urban Employee Basic Medical Insurance (UEBMI), Urban Resident Basic Medical Insurance (URBMI), and the New Rural Cooperative Medical Scheme (NRCMS)[11].Previous studies found that the proportion of screening in Liaoning was only 11.0%, far lower than the national average of 26.7%[12]. CC remains a major cause of morbidity and mortality among women in Liaoning due to low access to cancer screening. In the past ten years, the incidence rate of CC in Liaoning has risen from 18.05 to 100,000 to 21.82 per 100,000, and the mortality rate has increased from 4.14 to 100,000 to 6.27 per 100,000 million. High proportion of screening was essential to CC screening results[13]. The willingness of CC screening is closely related to the rate of CC screening, understanding factors that influence willingness of CC screening is important to increase the proportion of screening[14]. In our study, a population-based cross-sectional survey was conducted from 2018 to 2019 to analyze the factors of CC proportion of screening and the willingness of CC screening, we wanted to provide scientific basis for improving CC proportion of screening.

## Methods

### Study design and population

Our study was a cross-sectional survey which used the quantitative data collection methods. The study involved adult females who were habitually residents and aged from 30 to 69 years in Liaoning province, China. From all the county-level/district-level in Liaoning, we randomly selected nine county-level/district-level by random cluster samples (a convenience sampling method): Shenhe district, Tiexi district, Faku county, Lishan district, Benxi county, Qingyuan county, Xinglongtai district, Beizhen county and Zhuanghe county. We used a pretested semi-structured questionnaire to investigate the respondents through face-to-face inquiry, which facilitated participant understanding and completeness and thereby ensure the quality of the investigation.

This study was approved by the Medical Ethics Committee of Liaoning cancer hospital & institute (20,190,971), and confirmed the ethics guidelines of the Declaration of Helsinki. Informed consent was obtained from all participants before questionnaire administration.

### Data collection

The information of the questionnaire in our study contained socio-demographic characteristics, CC screening, previous screening history and the willingness of accepting CC screening. The questionnaire was filled out by uniformly trained investigators. All completed questionnaires were returned directly to the investigators. The variable definitions were as follow:

1. socio-demographic factors: age (30–39, 40–49, 50–59, 60–69); marital status (unmarried, married, others);
2. socio-economic factors: education level (below the junior middle school, senior middle school, college degree and above); medical insurance (medical insurance for urban employees/residents (MI), new rural cooperative medical scheme (NRCMS), others); type of occupation (working, retired, unemployed); family income (low-income: ≤ CNY10000, middle-income: CNY10000-50000, high-income: ≥CNY50000); health status (good, bad).
3. regional factors: the place of residence: according to the division code and urban-rural division code used by the National Bureau of statistics in 2018, nine survey sites were divided into urban and rural areas, the urban areas included: Lishan district, Shenhe district, Tiexi district, Zhuanghe county and Xinglongtai district; the rural areas included: Faku county, Beizhen county, Benxi county, Zhuanghe county. regional economic level: according to the relevant data in the statistical yearbook of Liaoning Province in 2018, by the two-point method, the per capita GDP (CNY) was divided into three categories: low level group (Faku county, Lishan district and Beizhen county), middle level group (Benxi county, Qingyuan county and Zhuanghe county), high level group (Shenhe district, Tiexi district and Xinglongtai district).
4. Previous screening history: The question was: Have you participated in cervical cancer screening in the past 3 years? If the respondents answered “yes”, the investigator should continue to ask about the examination fee, hospital level and examination quality.
5. The willingness of accepting cervical cancer screening: The question was: Are you willing to participate in cervical cancer screening in the next 3 years? If the respondents answered “yes”, the investigator should continue to ask about hospital level in which the respondent wanted to participate in.

### Statistical analysis

The project invited 700 women from each target area, 6300 women in total. Finally, 6077 people agreed to participate in the survey and fill in the relevant questionnaire. Before the survey process, all the investigators had received the unified training, we adopted the face-to-face investigation method. Data entry was conducted by the qualified personnel who also had received the unified training with the mode of back-to-back, and the consistency of the two entries had been compared by epidata software to check and correct the inconsistent data. In case of missing, logical error, unreasonable value and other records, the processing principle was to delete only the data points with errors, and kept other reasonable data points of the record. Before our statistical analysis, the data were further cleaned up, and the problems such as missing, logic errors and unreasonable values were further processed. Finally, 5334 records were reserved in the database for the relevant analysis of CC screening, with a response rate of 87.8%.

SPSS version 22.0 software (SPSS Inc. Chicago, IL) was used for statistical analysis. Multivariate logistic regression analysis was used to analyze the independent factors associated with the proportion of screening in the past three years and screening willingness in the next three years for different multivariate models. P < 0.05 (two-tailed test) was statistically significant.

## Results

### The characteristics of the respondents

Table 1 showed the characteristics of the 5334 respondents who participated in the survey. The average age of the women was 49.15 ± 11.16 years old. 90.70% of respondents were married women, 52.25% of respondents were below the junior middle school, 68.17% of respondents were working, 65.80% of respondents were urban employees/residents medical insurance, 61.23% of respondents were family income of 10,000–50,000 CNY/year, 59.12% of respondents were good in health status, 56.97% of respondents were urban residents, 35.47% of respondents were middle level in regional economic level. In the investigation on CC screening, 77.63% of respondents did not participate in CC screening in the past 3 years, and 61.59% of respondents did not have the willingness of CC screening in the next 3 years.

**Table 1:** The characteristics of respondents in our study

	N	(%)
Total	5334	100.00
Age		
30–39	1326	24.86
40–49	1397	26.19
50–59	1393	26.12
60–69	1218	22.83
Marital status		
Married	4838	90.70
Unmarried	136	2.55
Others	360	6.75
Education level		
Below junior middle school	2787	52.25
Senior middle school	1855	34.78
College degree and above	692	12.97
Type of occupation		
working	3636	68.17
retired/unemployed	1698	31.83
Medical insurance		
MI	3510	65.80
NRCMS	1421	26.64
Others	403	7.56
Family income		
Low-income	785	14.72
Middle-income	3266	61.23
High-income	1283	24.05
Health status		
good	3158	59.21
bad	2176	40.79
Place of residence		
Urban	3039	56.97
Rural	2295	43.03
Regional economic level (GDP per capita)		
Low level group	1733	32.49
Middle level group	1892	35.47
High level group	1709	32.04
Cervical cancer screening in past 3 years		
No	4141	77.63
Yes	1193	22.37
Willingness for cervical cancer screening in next 3 years		
No	3285	61.59
Yes	2049	38.41

MI, medical insurance; NRCMS, New Rural Cooperative Medical Scheme; GDP: Gross Domestic Product

### The effects of different factors on the respondents in the proportion of cervical cancer screening in the past 3 years

Table 2 showed the effects of different factors on the respondents in the proportion of CC screening in the past 3 years. Overall, the proportion of screening respondents was 22.37% in the past three years. The proportion of screening increased by age, the highest rate in the 40–49 age group was 31.07% (OR = 1.33, 95%CI: 1.13–1.58), and then decreased by age, the lowest rate in the 60–69 age group was 13.14% (OR = 0.45, 95%CI: 0.36–0.55). The proportion of screening in the unmarried group was lower than that in the married group (OR = 0.59, 95%CI: 0.37–0.95). The proportion of screening increased by education level, the highest rate in college degree and above was 35.98% (OR = 3.29, 95% CI: 2.73–3.97), the proportion of screening in retired/unemployed group was lower than that in working group (OR = 0.54, 95% CI: 0.47–0.63), and the proportion of screening in NCMS (OR = 0.44, 95% CI: 0.37–0.52) and others (OR = 0.73, 95% CI: 0.57–0.94) were lower than that in MI, the proportion of screenings in low-income group (OR = 0.50, 95% CI: 0.43–0.57) and middle-income group (OR = 0.40, 95% CI: 0.32–0.49) were lower than that in high-income group. The proportion of screenings in middle economic level group (OR = 2.65, 95% CI: 2.22–3.15) and high economic level group (OR = 2.54, 95% CI: 2.13–3.04) were higher than that of the low economic level group.

**Table 2:** The effects of different factors on the respondents in the rate of cervical cancer screening in past 3 years

	No. of cervical cancer screening(%)		Unadjusted			Multivariate Model 1			Multivariate Model 2			Multivariate Model 3		
			OR	(95%CI)	P value	OR	(95%CI)	P value	OR	(95%CI)	P value	OR	(95%CI)	P value
Age														
30–39	335	25.26	1.00			1.00			1.00			1.00		
40–49	434	31.07	1.33	(1.13, 1.58)	0.001	1.28	(1.08, 1.52)	0.004	1.37	(1.14, 1.63)	0.001	1.37	(1.14, 1.64)	0.001
50–59	264	18.95	0.69	(0.58, 0.83)	< 0.001	0.66	(0.55, 0.79)	< 0.001	0.86	(0.70, 1.05)	0.146	0.84	(0.69, 1.03)	0.100
60–69	160	13.14	0.45	(0.36, 0.55)	< 0.001	0.43	(0.35, 0.53)	< 0.001	0.64	(0.50, 0.81)	< 0.001	0.61	(0.48, 0.78)	< 0.001
Marital status														
Married	1094	22.61	1.00			1.00			1.00			1.00		
Unmarried	20	14.71	0.59	(0.37, 0.95)	0.031	0.50	(0.31, 0.81)	0.005	0.43	(0.26, 0.7)	0.001	0.40	(0.25, 0.67)	< 0.001
Others	79	21.94	0.96	(0.74, 1.25)	0.770	1.09	(0.83, 1.41)	0.544	1.05	(0.80, 1.37)	0.746	1.08	(0.82, 1.43)	0.573
Education level														
Junior middle school and lower	407	14.60	1.00						1.00			1.00		
Senior middle school	537	28.95	2.38	(2.06, 2.76)	< 0.001				1.66	(1.40, 1.97)	< 0.001	1.68	(1.41, 2.00)	< 0.001
College degree and above	249	35.98	3.29	(2.73, 3.97)	< 0.001				1.92	(1.52, 2.42)	< 0.001	1.86	(1.46, 2.36)	< 0.001
Type of occupation														
working	927	25.50	1.00						1.00			1.00		
retired/unemployed	266	15.67	0.54	(0.47, 0.63)	< 0.001				0.79	(0.66, 0.93)	0.006	0.79	(0.66, 0.94)	0.008
Medical insurance														
MI	920	26.21	1.00						1.00			1.00		
NRCMS	190	13.37	0.44	(0.37, 0.52)	< 0.001				0.65	(0.53, 0.79)	< 0.001	0.61	(0.49, 0.76)	< 0.001
Others	83	20.60	0.73	(0.57, 0.94)	0.015				0.82	(0.63, 1.07)	0.147	0.77	(0.59, 1.00)	0.053
Family income														
Low-income	128	16.31	0.50	(0.43, 0.57)	< 0.001				0.67	(0.57, 0.78)	< 0.001	0.69	(0.59, 0.81)	< 0.001
Middle-income	641	19.63	0.40	(0.32, 0.49)	< 0.001				0.69	(0.54, 0.88)	0.002	0.70	(0.55, 0.89)	0.004
High-income	424	33.05	1.00						1.00			1.00		
Health status														
good	733	23.21	1.00						1.00			1.00		
bad	460	21.14	0.89	(0.78, 1.01)	0.075				1.06	(0.92, 1.22)	0.395	1.03	(0.89, 1.18)	0.717
Place of residence														
Urban	685	22.54	1.00									1.00		
Rural	508	22.14	0.98	(0.86, 1.11)	0.725							0.36	(0.25, 0.51)	< 0.001
Regional economic level (GDP per capita)														
Low level group	217	12.52	1.00									1.00		
Middle level group	520	27.48	2.65	(2.22, 3.15)	< 0.001							1.76	(1.42, 2.18)	< 0.001
High level group	456	26.68	2.54	(2.13, 3.04)	< 0.001							3.41	(2.50, 4.66)	< 0.001

Multivariate Model 1: adjusted for socio-demographic factors (age and marital status); Multivariate Model 2: adjusted for socio-demographic factors (age and marital status) and socio-economic factors (education level, medical insurance, type of occupation, family income and health status); Multivariate Model 3: adjusted for socio-demographic factors (age and marital status), socio-economic factors (education level, medical insurance, type of occupation, family income and health status) and regional factors (Place of residence and Regional economic level)

MI, medical insurance; NRCMS, New Rural Cooperative Medical Scheme; GDP: Gross Domestic Product

The proportion of screening was further analyzed by multi-level logistic regression, which gradually added socio-demographic factors, socio-economic factors and regional factors. Compared to the results of univariate analysis, the proportion of screening in rural was lower than that in urban (OR = 0.36, 95% CI: 0.25–0.51), and the proportion of screening significantly increased by education level (P for trend = < 0.001), family income (P for trend = < 0.001) and regional economic level (P for trend = < 0.001).

### The effects of different factors on the respondents in the rate of willingness in cervical cancer screening in next 3 years

Table 3 showed the effects of different factors on the respondents in the rate of willingness on CC screening in next 3 years. Overall, the willingness rate of respondents was 38.41% in the next 3 years. The willingness rates in 50–59 age group (OR = 0.60, 95% CI: 0.51–0.71) and 60–69 age group (OR = 0.45, 95% CI: 0.38–0.53) were lower than that in 30–39 age group. The willingness rate increased by education level, the highest rate in college degree and above was 51.73% (OR = 2.31, 95% CI: 1.95–2.73), and the willingness rate in retired/unemployed group was lower than that in working group (OR = 0.69, 95% CI: 0.61–0.78), and the willingness rate in NCMS was lower than that in MI (OR = 0.67, 95% CI: 0.58–0.76). The willingness rates in the low-income group (OR = 0.56, 95% CI: 0.49–0.63) and middle-income group (OR = 0.61, 95% CI: 0.51–0.73) were lower than that in high-income group. The willingness rate in rural was higher than that in urban (OR = 1.15, 95% CI: 1.03–1.28), the willingness rates in middle economic level group (OR = 1.33, 95% CI: 1.16–1.52) and high economic level group (OR = 1.79, 95% CI: 1.56–2.06) were higher than that of the low economic level group. The willingness rate of respondents who had the experience of CC screening (OR = 7.23, 95% CI: 6.25–8.36) was higher than that in who had no experience of CC screening.

**Table 3:** The effects of different factors on the respondents in the rate of willingness on cervical cancer screening in next 3 years

	No. of willingness in cervical cancer screening(%)		Unadjusted			Multivariate Model 1			Multivariate Model 2			Multivariate Model 3			Multivariate Model 4		
			OR	(95%CI)	P value	OR	(95%CI)	P value	OR	(95%CI)	P value	OR	(95%CI)	P value	OR	(95%CI)	P value
Age																	
30–39	604	45.55	1.00			1.00			1.00			1.00			1.00		
40–49	647	46.31	1.03	(0.89, 1.20)	0.690	1.00	(0.86, 1.16)	0.970	1.01	(0.86, 1.18)	0.940	1.01	(0.86, 1.19)	0.870	0.90	(0.76, 1.07)	0.230
50–59	465	33.38	0.60	(0.51, 0.70)	< 0.001	0.58	(0.49, 0.67)	< 0.001	0.63	(0.53, 0.74)	< 0.001	0.63	(0.53, 0.75)	< 0.001	0.62	(0.52, 0.75)	< 0.001
60–69	333	27.34	0.45	(0.38, 0.53)	< 0.001	0.43	(0.37, 0.51)	< 0.001	0.49	(0.41, 0.60)	< 0.001	0.48	(0.40, 0.58)	< 0.001	0.50	(0.41, 0.62)	< 0.001
Marital status																	
Married	1873	38.71	1.00			1.00			1.00			1.00			1.00		
Unmarried	44	32.35	0.76	(0.53, 1.09)	0.134	0.59	(0.41, 0.85)	0.010	0.54	(0.37, 0.78)	< 0.001	0.56	(0.38, 0.81)	< 0.001	0.69	(0.47, 1.03)	0.070
Others	132	36.67	0.92	(0.73, 1.14)	0.441	1.03	(0.82, 1.29)	0.800	0.99	(0.79, 1.25)	0.950	1.03	(0.82, 1.30)	0.810	1.00	(0.78, 1.27)	0.970
Education level																	
Below junior middle school	884	31.72	1.00						1.00			1.00			1.00		
Senior middle school	807	43.50	1.66	(1.47, 1.87)	< 0.001				1.32	(1.15, 1.52)	< 0.001	1.32	(1.14, 1.53)	< 0.001	1.14	(0.98, 1.34)	0.090
College degree and above	358	51.73	2.31	(1.95, 2.73)	< 0.001				1.48	(1.20, 1.83)	< 0.001	1.48	(1.20, 1.83)	< 0.001	1.23	(0.98, 1.55)	0.070
Type of occupation																	
working	1496	41.14	1.00						1.00			1.00			1.00		
retired/unemployed	553	32.57	0.69	(0.61, 0.78)	< 0.001				0.94	(0.82, 1.07)	0.340	0.97	(0.84, 1.11)	0.630	1.04	(0.90, 1.21)	0.570
Medical insurance																	
MI	1442	41.08	1.00						1.00			1.00			1.00		
NRCMS	450	31.67	0.67	(0.58, 0.76)	< 0.001				0.81	(0.70, 0.95)	0.010	0.75	(0.63, 0.89)	< 0.001	0.85	(0.71, 1.02)	0.070
Others	157	38.96	0.92	(0.74, 1.13)	0.411				0.93	(0.74, 1.15)	0.490	0.89	(0.72, 1.11)	0.310	0.97	(0.76, 1.22)	0.770
Family income																	
Low-income	289	36.82	0.56	(0.49, 0.63)	< 0.001				0.68	(0.59, 0.78)	< 0.001	0.72	(0.62, 0.83)	< 0.001	0.79	(0.68, 0.92)	< 0.001
Middle-income	1133	34.69	0.61	(0.51, 0.73)	< 0.001				0.88	(0.72, 1.07)	0.200	0.90	(0.74, 1.10)	0.310	1.02	(0.82, 1.26)	0.870
High-income	627	48.87	1.00						1.00			1.00			1.00		
Health status																	
good	1191	37.71	1.00						1.00			1.00			1.00		
bad	858	39.43	1.08	(0.96, 1.20)	0.205				1.29	(1.15, 1.45)	< 0.001	1.30	(1.15, 1.46)	< 0.001	1.33	(1.17, 1.52)	< 0.001
Place of residence																	
Urban	1125	37.02	1.00									1.00			1.00		
Rural	924	40.26	1.15	(1.03, 1.28)	0.016							0.46	(0.36, 0.59)	< 0.001	0.57	(0.44, 0.74)	< 0.001
Regional economic level (GDP per capita)																	
Low level group	550	31.74	1.00									1.00			1.00		
Middle level group	722	38.16	1.33	(1.16, 1.52)	< 0.001							0.95	(0.8, 1.12)	0.530	0.78	(0.65, 0.94)	0.010
High level group	777	45.47	1.79	(1.56, 2.06)	< 0.001							2.30	(1.85, 2.85)	< 0.001	1.81	(1.44, 2.26)	< 0.001
Experience of cervical cancer screening																	
no	1167	28.18	1.00												1.00		
yes	882	73.93	7.23	(6.25, 8.36)	< 0.001										6.46	(5.51, 7.57)	< 0.001

Multivariate Model 1: adjusted for socio-demographic factors (age and marital status); Multivariate Model 2: adjusted for socio-demographic factors (age and marital status) and socio-economic factors (education level, medical insurance, type of occupation, family income and health status); Multivariate Model 3: adjusted for socio-demographic factors (age and marital status), socio-economic factors (education level, medical insurance, type of occupation, family income and health status) and regional factors (Place of residence and Regional economic level). Multivariate Model 4: adjusted for socio-demographic factors (age and marital status), socio-economic factors (education level, medical insurance, type of occupation, family income and health status), regional factors (Place of residence and Regional economic level) and Experience of cervical cancer screening

MI, medical insurance; NRCMS, New Rural Cooperative Medical Scheme; GDP: Gross Domestic Product

The willingness rate was further analyzed by multi-level logistic regression, which gradually added socio-demographic factors, socio-economic factors, regional factors and CC screening factors. Compared to the results of univariate analysis, in model 1–3, Age, marital status, education level, medical insurance, family income, health status, place of residence and regional economic level had a significant impact on willingness rate of CC. In model 4, Age, family income, health status, place of residence, regional economic level and experience of CC screening had a significant impact on willingness rate of CC.

## Discussion

In China, an estimated 28,010 women died from this preventable cancer, accounting for 12% of all CC deaths worldwide, CC is responsible for a heavy economic and social burden[15, 16]. CC is preventable and curable at early stages, screening of precancerous lesions can reduce its incidence and mortality. From the related results of the NORDCAN database, we found that the incidence of cervical cancer has been reduced by organized screening[17]. Therefore, CC screening is very important, and improving the proportion of screening of CC is helpful to reduce the incidence rate and mortality of CC. The World Health Organization (WHO) recommends that all women between the ages of 30 and 49 years should be screened for CC at least once[18]. In America, where CC screening programs have become well established, the annual incidence has declined by 75% or more over the past half century[19]. In our study, the proportion of screening in 2018 was 22.37%, which meant that more than three-quarters of women in Liaoning had not been screened for CC in the past 3 years. Though it was more than two times higher than 11.1% in 2013, it was still below 26.7% of the Chinese average proportion of screening, which was also still far below the proportion of screening in developed countries[12]. For example, the proportion of screenings amounted to 70% in Spain, 86.6% in Norway, 78% in UK, and 70% in Austria[20–22]. Women’s willingness to participate in CC screening had a direct impact on the screening work. Therefore, we further investigated the respondents’ willingness in the next three years, and found that the respondents’ willingness to participate in CC screening was only 38.41%. Even if all the people who had the willingness will participate CC screening in the next three years, the CC proportion of screening only increases by 16.04%. According to this increasing rate, it will take 12 years to achieve 100% of proportion of screening.

We divided the risk factors into socio-demographic factors, socio-economic factors, regional factors and CC screening factors, and gradually incorporate the risk factors into the regression model. In the factors of the proportion of screening, we found that age, marital status, education level, type of occupation, medical insurance, family income, place of residence and regional economic level had a significant impact on the proportion of screening. The proportion of screening significantly increased by education level, family income and regional economic level. In China, the free gynecological examination organized by the company is one of the main opportunities for women to participate in cervical cancer screening, women who had high education level might also have high family income and better occupations, and had more opportunities to participate in cervical cancer screening[23]. The proportion of screening was first increased and then decreased along with the increase of age. Although the proportion of screenings of CC in urban and rural areas were similar among the respondents, it seemed that the regional differences have no significant impact, but in multivariate logistic regression analysis, place of residence showed a significant impact on the proportion of screening, the proportion of screening in rural areas was 64% lower than that in urban areas. There were an estimated 570,000 new cases and 311,000 deaths from CC in 2018, and around 85% of these occurred in low-income countries [24]. In China, the incidence rate for CC was about 10% higher in rural areas (12.35 per 100,000) than in urban areas (11.24 per 100,000), and the mortality rate was about 12% higher in rural areas (3.50 per 100,000) than in urban areas (3.12 per 100,000)[25]. The disease burden was heavier in rural areas than in urban areas, therefore, it is imperative to improve the proportion of screening of CC in rural areas.

In the factors of the screening willingness, we found that in model 1–3, age, marital status, education level, medical insurance type, family income, health status, place of residence and regional economic level had a significant impact on the screening willingness. Compared to the analysis on the factors of the proportion of screening, the type of occupation had no significant impact on the screening willingness. After the experience of CC screening was added in model 4, age, family income, health status, place of residence, regional economic level and CC screening still had a significant impact, but marital status, education level and medical insurance type had no significant impact. There was no significant difference in marital status, education level and medical insurance type after the factors of CC screening were added in model 4. This shows that after participating in CC screening, the respondents may learn more about the relevant knowledge of CC screening and pay more attention to the changes of the body. Therefore, we think it is very important to participate in CC screening for the first time in the future.

Age had a significant impact on the proportion of screening and willingness of CC. Young women, especially those with higher education level and/or higher income level, were likely to pay more attention to their health and had more access to relevant information to increase their knowledge[26]. There were some differences in the factors between the CC proportion of screening and the willingness of CC screening. Type of occupation had a significant impact on CC proportion of screening, but had no significant impact on the willingness of CC screening, people at work were more likely to participate in CC screening, and retiree or people without work were less likely to participate in CC screening. Physical examination is the main way for women to get CC screening, while people without work have few chances to get screening through physical examination by the occupational way. However, our study found that the proportion of screening of working women was only 25.50%, which reminded us that CC screening had not been well included in physical examination, and had a very limited effect on the prevention and control of CC in the population. Especially in small and medium-sized and private enterprises, physical examination belonged to the spontaneous behavior and had a certain market attribute. The specific implementation of physical examination including content, frequency and quality was not optimistic[27, 28]. In addition, the opportunity for retired women to receive CC screening was reduced. In China, the retirement age of women is mainly 55 years old, and they are still in the important age for CC screening[29]. At the same time, they are in the age of high CC mortality rate[30, 31]. Therefore, this part of the population should be considered in future screening.

Health status has a significant impact on the willingness of CC screening, but has no significant impact on CC screening. The coverage of screening might be still insufficient, and the willing women may not be able to access the screening due to potential barriers.[32, 33] Family income was an important socio-economic factor to promote women’s CC screening, which should be considered in improving the coverage of CC screening in China. The effect of family income on the proportion of screening emphasized that providing free screening services would have a positive significance in improving the current CC proportion of screening in China. At the same time, we should provide affordable and low-cost screening services for more people to accept screening voluntarily.

Regional factors, which included place of residence and regional economic level, were also the important factors that affect the proportion of screening and willingness of CC. Urban areas and regions with higher economic level usually had more infrastructure, medical and health system, and higher level of medical and health personnel, so CC screening services in these regions have higher accessibility. In China, the county-level/district-level institutions were the main prescreening institutions for CC screening in NCCSP. A nationwide study found that in terms of facilities, medical staff and equipment, medical institutions in urban areas can provide better CC screening equipment in general than the institutions in rural areas, a relatively large gap still exists in the current health service capacity between urban and rural areas[34].

Health education programs which aim to increase the understanding of CC and learn the benefits of screening had been found to be an effective method to increase screening willingness, as well as increase the likelihood of screening behaviors[35]. Previous studies had found that due to the low education level, a portion of women did not understand and/or were not willing to read the CC information booklet provided by the government. They preferred to attend free face-to-face seminars on CC prevention and control provided by health care workers, or watch relevant health promotion videos[36]. A multimedia health education program has been found to effectively improve the knowledge and awareness of CC and its screening, and also increased the uptake of screening services among adult rural women in Nigeria[37].

There were also some shortcomings in our study. First, we only selected counties/districts in Liaoning Province, this might make our extrapolation of results have a certain regional limitation. Nevertheless, we randomly selected 9 counties/districts in Liaoning Province, and grouped them according to different regions and different levels of economic development, which could fully reflect the situation of different people participating in cervical cancer screening and their willingness in the local regions, and could provide a certain reference basis for research in other regions. Second, in European countries, according to the questionnaire, there were large variations in cervical cancer screening policies, and the key organizational elements of the plan were also insufficient[38]. Compared to the research contents in European countries, our research paid too much attention to the common factors of the screening women, and did not involve the minimum requirements, the fairness and accessibility of the cervical cancer screening. We will further explore the relevant contents in the future study.

## Conclusion

CC screening is an effective way to reduce the incidence and mortality rate of CC. It is imperative to improve the proportion of screening of CC in China. Our study found both proportion of screening and willingness were at a low level, and age, economic and regional factors were the main factors for implementation of CC screening in China. In the future, in order to improve the proportion of screening of CC, targeted policies should be formulated according to the characteristics of different groups of people, for example health education strategies, and reduce the gap in the current health service capacity between different regions.

## Data Availability

The datasets used and/or analyzed during the current study available from the corresponding author on reasonable request.
